# Investigation of the Involvement of HHV-6 Encoded Viral Chemokine Receptors in Autoimmune Thyroiditis Development

**DOI:** 10.1128/spectrum.02369-21

**Published:** 2022-05-23

**Authors:** Alina Sultanova, Maksims Cistjakovs, Liba Sokolovska, Egils Cunskis, Modra Murovska

**Affiliations:** a Riga Stradins University, Institute of Microbiology and Virology, Riga, Latvia; b Riga East Clinical University Hospital, Clinic “Gailezers”, Riga, Latvia; Oklahoma State University, College of Veterinary Medicine

**Keywords:** autoimmune thyroiditis, HHV-6, G protein-coupled receptors, chemokine receptor, antibodies

## Abstract

Human herpesvirus-6 (HHV-6) contains two genes (U12 and U51) that encode putative homologues of human G-protein-coupled receptors like CCR1, CCR3, and CCR5. It has been shown that these viral proteins can be expressed on the surface of epithelial and some peripheral blood mononuclear cells, suggesting that they could potentially induce autoimmunity. We aimed to investigate the possibility of HHV-6 encoded viral chemokine receptors (U12 and U51) involvement in autoimmune thyroiditis (AIT) development by detecting viral peptide specific antibodies in AIT patient samples. Seventy-nine AIT patients whose thyroid tissues were shown to be positive for HHV-6 and 32 blood donors were enrolled in this study. Twenty-eight synthetic peptides derived from HHV-6 U12 and U51 proteins’ amino acid sequences, as well as recombinant human CCR1, CCR3, and CCR5 proteins were used in suspension multiplex immunological assay to detect specific IgG and IgM antibodies. HHV-6 peptide specific IgG and IgM antibodies were found in patients’ samples. AIT patients' samples were found to be more frequently positive for peptide IgGs in comparison to control group’s samples. Even though peptide antibody cross-reactivity with human CCRs was not demonstrated, our results show a new immunogenic HHV-6 antigen—a possible new player in the HHV-6 induced autoimmunity exacerbation.

**IMPORTANCE** The study of human herpesvirus-6 (HHV-6) involvement in autoimmunity development is very challenging, due to the complex nature of this virus. HHV-6 is a ubiquitous, lifelong persistent, and immunomodulating virus, which mainly spreads in solid tissues using cell-to-cell mechanics, and thus can escape from the host’s immune response. It has been implicated as an environmental factor in several autoimmune diseases. An association between HHV-6 and autoimmune thyroiditis has been demonstrated, yet clear mechanism of involvement remains to be elucidated, since the virus can be detected in nearly all autoimmune thyroiditis patient thyroid glands. Our results show new potentially immunogenic human herpesvirus-6 antigens—possible new players in the HHV-6 induced autoimmunity exacerbation, which could be subjects for further research. Together with previously published results, this study described possible mechanisms which may underlie the induction of autoimmune reactivities against thyroid tissues in AIT.

## INTRODUCTION

As Human herpesvirus-6 (HHV-6) is able to establish lifelong latency and reactivate, the infection can influence its hosts health beyond the primary infection and can contribute to the development of several autoimmune disorders, including autoimmune hemolytic anaemia/neutropenia (1), autoimmune acute hepatitis (2), and multiple sclerosis (3–5). Recently, more and more attention has been given to the possible involvement of HHV-6 in the development of autoimmune thyroiditis.

One of the recently published studies link HHV-6 to Hashimoto thyroiditis (HT) (6). This study demonstrates that thyroid fine needle aspirates (FNA) obtained from patients with HT revealed the presence of HHV-6 significantly more frequently in comparison with the controls (82% and 10%, respectively). Furthermore, active HHV-6 transcription is observed in HT thyrocytes, compared with the latent infection found in thyroid tissue samples used as a control. These researchers propose a potential mechanism for HHV-6-induced autoimmunity demonstrating that follicle cells infected with HHV-6 became susceptible to NK-mediated killing (6). Also, our previously published data show an almost 100% incidence rate of HHV-6 genomic sequence in thyroid gland tissue samples acquired from autoimmune thyroiditis (AIT) patients' post-surgical materials (7). Furthermore, AIT patients' thyroid tissue samples were significantly more frequently positive for HHV-6 activation marker (HHV-6 U79/80 mRNA) in comparison to the control group (18/44 [41%] versus 1/17 [6%], respectively; *P* = 0.0118) (7).

Even though based on clear epidemiological, immunological and molecular differences two HHV-6 variants have been recognized as two distinct viruses (HHV-6A and HHV-6B), it is not clear which one of them is implicated in AIT development (8). Different patterns in the distribution of both viruses have been observed. The Italian group that linked HHV-6 to Hashimoto thyroiditis reports the presence of HHV-6A in their patients’ tissues samples, but our previous investigations show the presence of HHV-6B in AIT patients’ samples (6, 7).

HHV-6 possesses a number of immunomodulating properties for immune evasion and viral dissemination. These include the ability to alter the repertoire of molecules expressed on infected cell surfaces, as well as chemokine and cytokine expression and secretion modulation (9). Another immunomodulation strategy utilized by herpesviruses, including HHV-6, is the ability to encode viral chemokines and chemokine receptors (10).

HHV-6 encodes two viral chemokine receptors U12 and U51, which are structurally similar to cellular G protein-coupled receptors (GPCR) (11), but the role of these proteins is not well understood. Although the HHV-6 gene encoding the chemokine U83 is highly variable between the species of HHV-6 resulting in different chemokine binding specificities, the two chemokine receptors are highly similar, sharing amino acid identity of up to 99% between the two viruses (10). HHV-6 U12 and U51 are functionally similar to the much better studied Cytomegalovirus (CMV) protein US28 (pUS28), which enhances the course of CMV infection. As a CCR homologue, pUS28 dimerizes with a number of host chemokine receptors, including CCR1, CCR2, CCR3, CCR4, CCR5, and CXCR4 (12). HHV-6 encoded GPCRs are not sufficiently studied, which raises the question of whether these proteins are involved in the development of autoimmune processes and how it would affect the host's immune system if they are able to dimerize with the HHV-6 “chemokine receptors” in a host similar to the CMV protein US28.

It has been shown that proteins which are encoded by HHV-6 U12 and U51 genes can be expressed on the surface of epithelial and some peripheral blood mononuclear cell populations, suggesting that these viral proteins could serve as potential antigens and in solid tissue settings could enhance inflammation or may be even play a role as trigger for autoimmune response (13, 14). Additionally, it has been shown *in vitro* that HHV-6 encoded GPCRs could interact with cytokine signaling pathways by downregulating RANTES (15). A recent study we published demonstrates a relatively high frequency of HHV-6 U12 and U51 mRNA presence in AIT patients' thyroid gland tissue samples, as well as significantly lower RANTES levels in peripheral blood in comparison to blood donors (16).

Unfortunately, both HHV-6 encoded chemokine receptors are poorly studied and studies that have been conducted were done more than 20 years ago, which means their role in HHV-6 infection and disease development remains unclear. One of the reasons why chemokine receptors encoded by HHV-6 are poorly studied is the trans-membrane nature of these proteins and the difficulty in purifying them for recombinant protein acquirement. To acquire antigens, which could be effectively used for immunization and investigation of their possible involvement in autoimmune diseases development, synthetic peptides from HHV-6 U12 and U51 amino acids sequences are designed in this study. Even though the presentation of potential linear epitopes in the form of synthetic peptides cannot represent the full antigenicity of a whole protein, it provides a much cheaper and faster approach for the research of immunogenicity of these HHV-6 proteins.

Knowing the structural and functional similarity of HHV-6 U12 and U51 proteins with cellular GPCRs and their ability to be expressed on cell surfaces, their role in autoimmunity development is worth considering. Therefore, the aim of this study was to investigate the immune response in AIT patients to HHV-6 encoded viral chemokine receptors (U12 and U51) by detecting viral peptide specific antibodies in AIT patient samples and to determine whether antibodies specific for HHV-6 chemokine receptors can cross-react with human chemokine receptors CCR1, CCR3, and CCR5 based on their structural and functional similarities.

## RESULTS

### Antibodies specific to synthetic peptides detected by suspension multiplex immunoassay (SMIA).

AIT patients and blood donors’ plasma pools were used for initial run to find immunogenic peptides. From 28 synthetic peptides—10 peptides showed high MFI values and were taken for further investigation (Fig. S3 in the supplemental material). Eight synthetic peptides (peptides designed from HHV-6 U12: HHV-6_GPCR4; HHV-6_GPCR6; HHV-6_GPCR7; HHV-6_GPCR12 and HHV-6_GPCR15, and from HHV-6 U51: HHV-6_GPCR24; HHV-6_GPCR26 and HHV-6_GPCR27) showed high MFI values for IgG and the same peptides + two more for IgM (for HHV-6 U12: HHV-6_GPCR13, as well as for HHV-6 U51: HHV-6_GPCR22). To illustrate that the high IgM signals obtained were truly of peptide specific IgMs, IgG/Rf sorbent was used and even after the use of IgG/Rf absorbent peptides showed significantly high IgM MFI signals (Figs. S3–S4 in the supplemental material).

After the run with all plasma samples from both groups together with preabsorbed samples using immunogenic peptides there was a need of additional data analysis, as some positive signals were very close to NAC signals (Fig. S5 in the supplemental material). Therefore, negative samples’ mean MFI + 2 SD for each peptide was used as an additional cut off to get rid of very low (unspecific) MFI values.

### IgG antibodies specific to synthetic peptides detected by SMIA.

**FIG 1:**
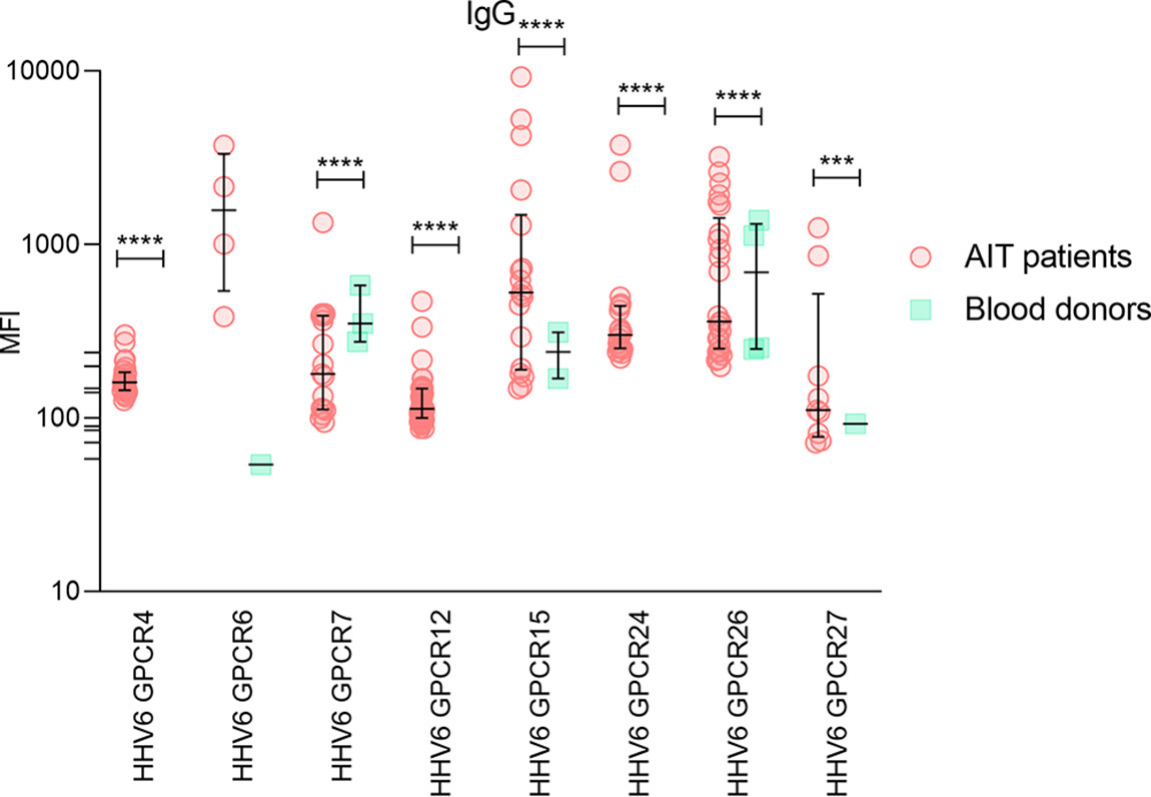
SMIA results of IgG antibodies against 8 immunogenic HHV-6 synthetic peptides in AIT patients’ and blood donors’ groups (*, significant difference).

Of the 8 immunogenic synthetic peptides tested for specific IgG class antibodies the highest median fluorescent signal was shown by HHV-6_GPCR6 peptide (designed from HHV-6 U12 protein). This peptide demonstrated a non-significantly higher MFI signal in AIT patients when compared with BD (MFI 1582.0; inter-quartile range [IQR]: 540.1-3346.0 vs MFI 63.75; [IQR] 54.0–73.5, *P* = 0.0850) as determined by the Mann-Whitney U test (Fig. 1; IgG). However, all of the other peptides analyzed showed significantly higher MFI values (all *P* < 0.0001, expect one HHV-6_GPCR27, which was showing *P* = 0.0002) when comparing patients with BD group (excluding the peptides, to which no positive MFI values were obtained in BD and comparison was not done).

Moreover, among groups, AIT patient group’s samples were more frequently positive for HHV-6 peptide IgGs in comparison to BD group’s (Table 1). Significantly (using Chi square test) more frequent IgG antibody presence was found against 5 HHV-6 synthetic peptides: three corresponding to HHV-6 U12 (GPCR4, GPCR12 and GPCR15) and two (GPCR24 and GPCR26) corresponding to HHV-6 U51 (Table 1). Interestingly, IgG antibodies against GPCR4, GPCR6, GPCR12 and GPCR24 peptides were found only in AIT patients’ group (Fig. 1; Table 1).

**TABLE 1:** Frequency of IgG antibodies presence against HHV-6 immunogenic peptides in AIT patients’ and blood donors’ groups

Peptides	AIT patients (*n* = 79)	Blood donors (*n* = 32)	*P*
HHV6_GPCR4	25 (32%)	0 (0%)	<0.001
HHV6_GPCR6	4 (5%)	0 (0%)	0.634
HHV6_GPCR7	17 (22%)	3 (9%)	0.131
HHV6_GPCR12	41 (52%)	0 (0%)	<0.0001
HHV6_GPCR15	18 (23%)	2 (6%)	0.040
HHV6_GPCR24	19 (24%)	0 (0%)	0.020
HHV6_GPCR26	25 (32%)	4 (13%)	0.037
HHV6_GPCR27	9 (11%)	1 (3%)	0.168

### IgM antibodies specific to synthetic peptides detected by SMIA.

**FIG 2:**
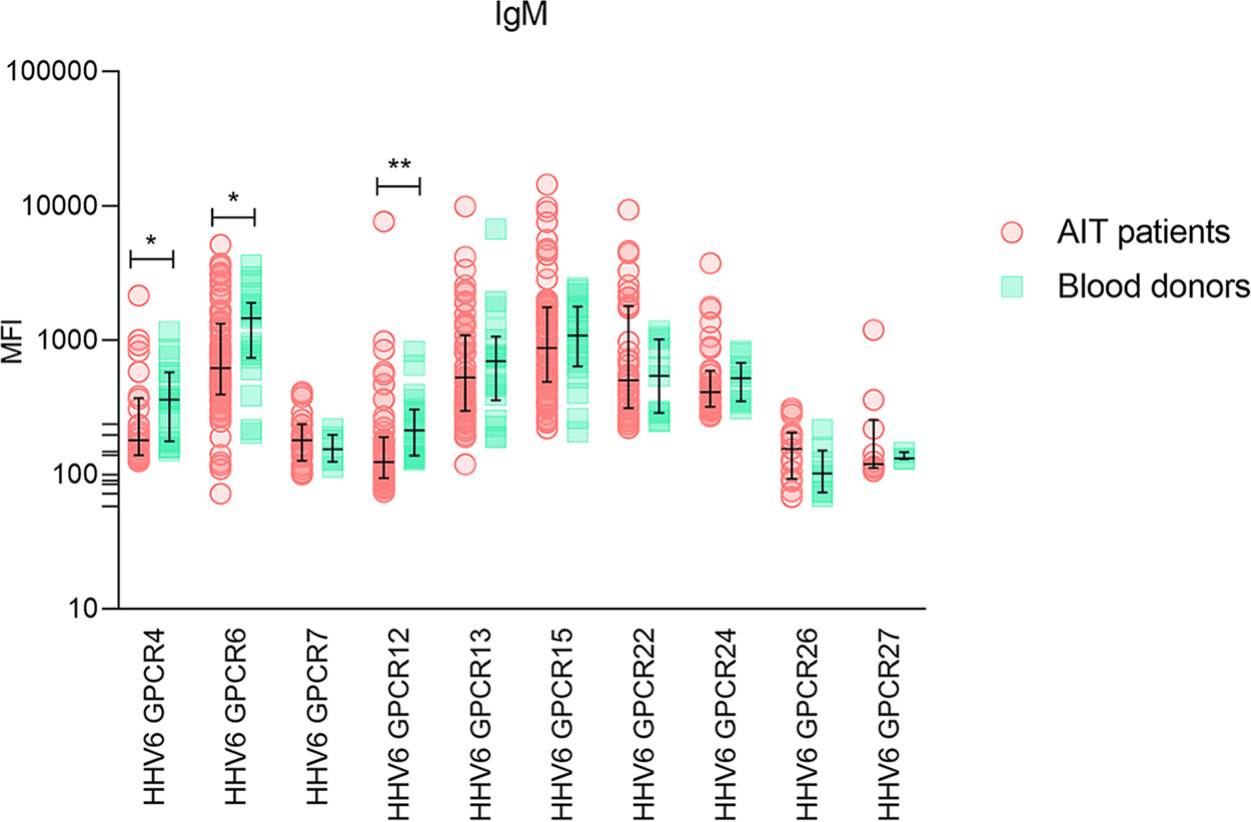
SMIA results of IgM antibodies against 10 immunogenic HHV-6 synthetic peptides in AIT patients’ and blood donors’ groups (*, significant difference).

Of the 10 peptides tested in both groups for specific IgM class antibodies the strongest median fluorescent signal (MFI 1207; IQR: 722.8 - 1837) was shown by HHV-6_GPCR6 peptide in blood donors’ group (Fig. 2). Overall, Mann-Whitney test showed significantly (*P* < 0.05) higher levels of IgMs against 3 out of 10 synthetic peptides in blood donors’ group (Fig. 2). Like in the case of IgG, two peptides—HHV-6_GPCR4 and HHV-6_GPCR12 (peptides derived from HHV-6 U12), showed significant difference between AIT patients and blood donors.

Comparing peptides positive for IgG and IgM antibodies, the most visible difference could be observed in the number of positive peptides per individual. In IgG investigations, 3–5 peptides positive per individual was the highest number, while for IgM it was 7–10 peptides. Significant difference in number of positive peptides per individual was found between AIT patients and blood donors, in contrast, there was minimal difference found in the case of IgM positive peptides (Table 2).

**TABLE 2:** Simultaneous multiple peptide IgG or IgM antibody detection in AIT patients and blood donors

IgG				IgM			
No. of positive peptides	AIT patients (*n* = 79 [%])	Blood donors (*n* = 32 [%])	*P*	No. of positive peptides	AIT patients (*n* = 79 [%])	Blood donors (*n* = 32 [%])	*P*
3 or more	25 [32%]	0 [0%]	0.001	7–10	22 [28%]	11 [34%]	0.495
Two	18 [23%]	2 [6%]	0.040	5–6	26 [33%]	11 [34%]	0.872
One	20 [25%]	8 [25%]	0.972	3–4	28 [35%]	1 [3%]	<0.001

The most common combination of IgG peptides for HHV-6 U12 were: HHV-6_GPCR4 and HHV-6_GPCR12 (24/79 [30%]); for HHV-6 U51: HHV-6_GPCR24 and HHV-6_GPCR26 (14/79 [18%]).

### IgG and IgM antibodies specific to human recombinant protein CCR1, CCR3, and CCR5 detected by SMIA.

Beads coupled with human chemokine receptors (CCR1, CCR3, and CCR5) were incubated with pooled patient plasma samples to determine whether AIT patients have potential auto-antibodies against human GPCRs. The highest MFI values of IgG antibodies were observed in the case of CCR1 (MFI 319.5, IQR: 252.5—429.5) and the highest MFI values of IgM antibodies were detected for CCR5 (MFI 1420, IQR: 412.8—4907; Fig. S6 in the supplemental material).

Comparison of median MFI values did not show any significant difference between AIT patients and blood donors’ group, both in IgG and IgM levels against recombinant CCRs (Fig. S6 in the supplemental material).

### Analysis of HHV-6 load and antibodies against HHV-6 peptides detected by SMIA.

**FIG 3:**
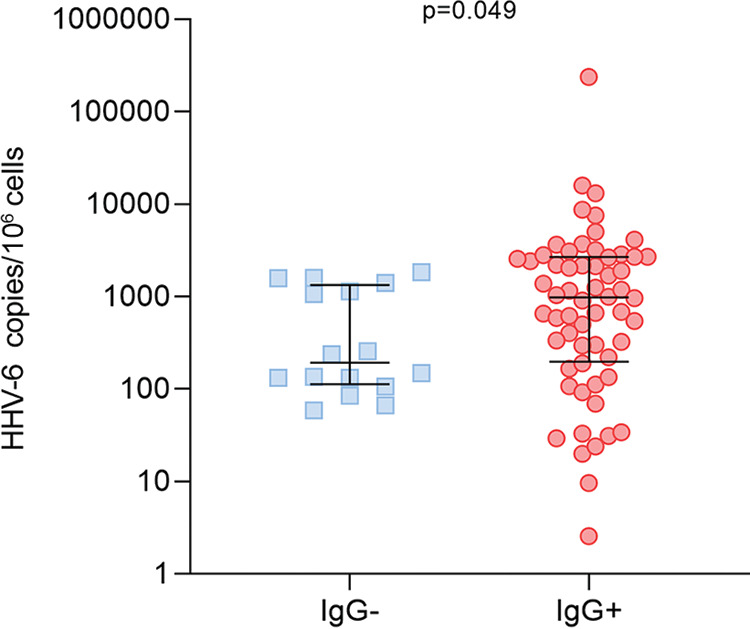
HHV-6 load in thyroid gland tissues found in AIT patients negative and positive for synthetic peptide IgGs.

In addition, we used information about HHV-6 viral load in thyroid tissue samples of the AIT patients analyzed in this study. Using Mann-Whitney U test, we found that AIT patients, who were shown to be positive for any of the synthetic peptide IgGs, have significantly higher (*P* = 0.049) median HHV-6 load than AIT patients, who were negative (978.8 [IQR: 197.4–2677] viral copies/10^6^ cells vs 193.3 [IQR: 113.0–1333.0] viral copies/10^6^ cells; Fig. 3).

In its turn, comparison of antibodies levels against human CCR1 and CCR5 (the most reactive proteins) between AIT patients with low and high HHV-6 load (<200 and >200 copies/10^6^ cells, respectively) in thyroid gland showed no significant difference (Fig. S7 in the supplemental material).

### Analysis of thyroid auto-antibodies and antibodies against HHV-6 peptides detected by SMIA.

**FIG 4:**
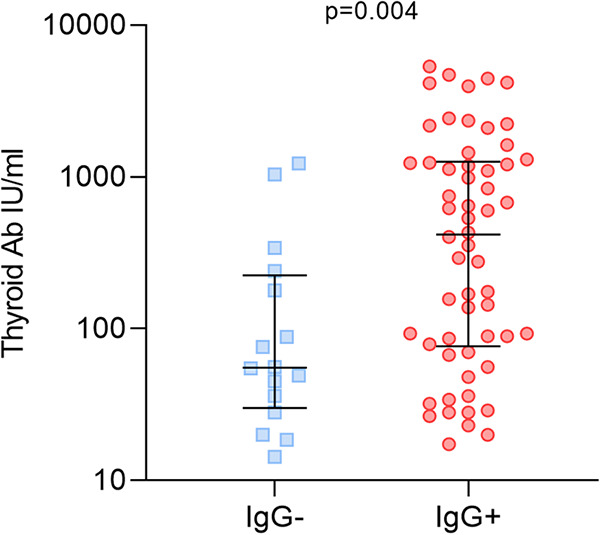
Thyroid auto-antibody levels (TPO or TG) in AIT patients negative and positive for synthetic peptide IgGs.

Also, we were able to compare AIT patients’ thyroid gland auto-antibodies levels in synthetic peptide IgG positive and IgG negative patients. Using Mann-Whitney test, median level of thyroid auto-antibody (TPO either TG) antibody was found significantly higher (*P* = 0.004) in AIT patients positive for viral peptide IgGs in comparison with patients who were negative (416.2 [IQR: 76.75—1257] IU/mL vs 55.36 [IQR: 30.0–224.8] IU/mL; Fig. 4). Although, sufficient difference between U12 and U51 peptides was not found (680.0 [IQR: 36—1307] IU/mL vs 534.0 [IQR: 169.3—1620] IU/mL).

Separately by the peptide, we could not find any correlations neither with HHV-6 activity nor auto-antibody levels, however, IgG antibodies against U12 peptides were found in AIT patients more frequently than against U51 peptides (*n* = 24 vs *n* = 8).

### Assessment of HHV-6 peptide antibody cross-reactivity with human recombinant CCRs.

AIT patients’ and blood donors’ plasma pools did not show any significant signal changes after preabsorbtion with either of the human recombinant proteins (CCR1, CCR3, and CCR5). No pronounced differences or no changes at all were observed even in the signals of the most immunogenic peptides (HHV-6_GPCR6 e.g.), indicating that the linear epitopes analyzed in this study were not able to induce antibody cross-reactivity with human CCRs (Figs. S8–S9 in the supplemental material).

The preabsorbtion of reactive plasma pools with peptides, also did not significantly affect CCR IgG and IgM MFI signals (Fig. S10 in the supplemental material).

## DISCUSSION

HHV-6 is a widely distributed human pathogen, which has been implicated in a number of human diseases, many of them autoimmune. The role of HHV-6 in autoimmune thyroid disease is still unclear. Previous studies showed high frequency of HHV-6 genomic sequence presence in thyroid gland samples obtained from patients with AIT, some have even proposed autoimmunity triggering mechanisms, but not with undeniable certainty (17–19).

Results from our own and other studies led us to believe that the chemokine receptors encoded by HHV-6 (U12 and U51) could be involved in the development of AIT. Firstly, we have observed that the chemokine receptors are expressed in the thyroid gland tissues of AIT patients and that the levels of chemokine RANTES, which can be bound and downregulated by the viral chemokine receptors (15), are significantly decreased in AIT patients plasma samples (16). Secondly, U12 and U51 can be expressed on infected cell surfaces and they possess structural and functional similarities with human chemokine receptors (15).

Another reason why specifically these two HHV-6 proteins were chosen for closer study is that commercial ELISAs were not informative in our studies, and we used them only for seropositivity confirmation. The majority of commercial kits use viral capsid or capsid proteins as antigens, meaning that significant HHV-6 IgG and IgM titer changes could only be observed during primary and acute infection, when a lot of viral particles are exposed to the immune system (20). This is not always the case, as HHV-6 can persist in solid tissues and use mainly cell-to-cell spread tactics like other herpesviruses (21). Such spread combined with immunomodulating properties of HHV-6 ensures its lifelong persistence within the host. In previous studies we also showed dominance of HHV-6 activity in thyroid tissue and the absence of it in peripheral blood (7, 16). Since U12 and U51 can be expressed on infected cell’s surface, these proteins could be more frequently exposed to the immune system, than for example, major capsid protein, as virus remains in solid tissues during the cell-to-cell spread. While spreading in this manner there is no need to produce a lot of viral particles to infect other cell, which is supported by previous studies, where HHV-6 loads were found relatively low in tissues with active viral markers (7, 16).

Goals of our study included the detection of HHV-6 U12 and U51 specific antibodies and the determination whether these antibodies can cross-react with human chemokine receptors CCR1, CCR3 and CCR5, based on their structural and functional similarities, like the expression on cell surfaces and the ability to bind RANTES. Since U12 and U51 are transmembrane in nature, it was decided to use synthetic peptides designed from viral proteins' amino acids. Use of synthetic peptides has its pros and cons. The main pro is that they are cheap to produce and they can contain specific linear epitopes which can be used for diagnostic purposes or for the acquirement of specific antibodies. The absence of full immunogenic information, for example, conformational epitopes, can be considered as the main con.

Synthetic peptides used in this study were designed based on the analysis of two parameters - conservative regions from alignment with potential human homologs (CCR1, CCR3, and CCR5) and predicted epitopes (using Bepipred Linear Epitope Prediction and Kolaskar & Tongaonkar algorithms). This approach was chosen to increase the chance of acquiring immunogenic peptides and was meant to be more cost efficient for the implementation. However, this approach does not guarantee the possibility of analyzing full linear epitopes. To fully describe a particular linear epitope and its immunogenicity, further research should be done using overlapping amino sequences of peptides which were immunogenic in this study.

Both peptide specific IgG and IgM antibodies were found in AIT patients’ and blood donors’ plasma samples, but the most important finding of this study is the predominance of peptide specific IgG antibodies in AIT patients’ plasma, comparing with blood donors. It is known that the majority of antigens that bind B cell receptors are self-antigens, but maturation of self-reacting clones is restricted by central and peripheral tolerance mechanisms (22, 23). The IgG/IgM differences observed in our study between AIT patients and blood donors, could be in part explained by the breakdown of immunological tolerance observed in autoimmunity. While in blood donors these potentially autoreactive B cells are not allowed to mature to IgG-producing cells, they may be allowed to mature in autoimmunity patients, thus explaining the predominance of IgGs in AIT patients (23). This explanation is supported by several studies that suggest that IgG auto-antibodies usually play an important role in autoimmune diseases development (24–26). Furthermore, the significantly higher IgM levels against these peptides found in blood donors’ plasma samples could indicate on normal immune system function. Studies in systemic lupus erythematosus mice models, demonstrated that mice unable to secrete IgM antibodies developed autoimmune disease faster and with more severe symptoms than mice with induced IgM expression (24). These findings demonstrate that secreted IgM can suppress the development of IgG auto-antibodies and autoimmune disease under physiological conditions, showing important role of IgM antibodies in balancing of immune response.

Another explanation of the differing antibody reactivities may be associated with HHV-6 infection itself. As the HHV-6 infection is very common worldwide, and this virus persists within the host, it could reactivate often—which can explain high levels of IgM found in blood donors. Alternatively, since us and other researchers have demonstrated that HHV-6 is highly prevalent and more active in AIT patients, the higher frequency and high levels of IgG antibodies against HHV-6 peptides indicate a continuous immune stimulation by HHV-6 and these two specific viral proteins and thus could indicate the possible involvement of HHV-6 U12 and U51 in autoimmunity development.

To further analyze the relevance of the presence of synthetic peptide specific IgGs in the context of thyroid infection by HHV-6, we used molecular data on HHV-6 presence in AIT patients and immunologic data on thyroid auto-antibody published in our previous article (16).

The presence of HHV-6 U12 and/or U51 mRNA was found in 34 out of 79 (43%) AIT patients’ thyroid gland. What is interesting, 30 out of 34 AIT patients positive for HHV-6 chemokine receptors mRNA in their thyroid gland, were also positive for HHV-6 U12 and/or U51 synthetic peptide specific IgGs. In contrast, 30 autopsied subjects (26 women and 4 men; median age 58; IQR: 51–67) without thyroid pathologies, who were included as a control group in our previous research, showed the absence of any HHV-6 U12 and/or U51 mRNA in their thyroid gland tissue samples (16). For obvious reasons, it is impossible to show HHV-6 chemokine receptors mRNA presence in blood donors’ thyroid gland tissues. Therefore, we can only assume that presence of viral mRNA in healthy individuals’ thyroid gland is near to zero. It is worth to mentioning that HHV-6 DNA was found only in 10% of blood donors’ peripheral blood samples by nested-PCR method and the viral load was not even detectable by qPCR technique. Interestingly, IgG antibodies against HHV-6_GPCR4, HHV-6_GPCR6, HHV-6_GPCR12, and HHV-6_GPCR24 peptides were found only in AIT patient samples, highlighting the need for further investigations including more patients, and further amino acid sequence analysis, to evaluate whether these specific peptides have a role in autoimmunity development.

In addition, dominant presence of IgG antibodies to 8 peptides was shown to be associated with higher HHV-6 activity (both viral load and presence of HHV-6 U12 and U51 mRNA) in AIT patients’ thyroid gland and with higher levels of thyroid gland auto-antibodies. This indicates that there may be a connection between viral activity in the thyroid, the immune response against it and the pathogenesis of AIT - the production of thyroid antibodies. Productive viral infection promotes the destruction of thyroid gland directly and thus promotes the exposure of thyroid antigens and the subsequent anti-thyroid auto-antibody production. Although, it’s worth mentioning that the opposite may also be true—that in patients with high thyroid auto-antibody titers, where thyroid destruction occurs, not only self-antigens are released but viral antigens also, meaning that thyroid destruction could enhance the production of anti-HHV-6 antibodies, thus explaining the high viral IgG presence accompanied by high thyroid auto-antibody titers. If the latter indeed could occur and HHV-6 antibodies are indeed capable of autoimmune process exacerbation, such a scenario would lead to an inflammatory feedback loop where anti-thyroid antibodies lead to thyroid destruction, native and viral antigen release and in its turn to increased inflammation by immune responses responding to both native and viral antigens.

In addition, dilution assay of reactive AIT patients’ and blood donors’ plasma, which was done to evaluate approximate quantity of antibodies, shows undetectable signals from all peptides - both for IgG and IgM at 1:400 dilution. At 1:200 dilution more frequent positives are detected for IgM antibodies (10/10 vs 3/10, respectively) in patients’, as well as in donors’ samples. Such low titers were expected as this study uses synthetic peptides, which involves the analysis of antibody fractions recognizing only specific linear epitopes. Peptide specific antibody fractions have been demonstrated to be significantly smaller in comparison to antibody fractions recognizing conformational epitopes and using only linear epitopes does not illustrate the full immunogenic picture (27). This shows that the use of recombinant proteins would show higher titers than using peptides, as these recombinant proteins could bind conformational epitope and some linear epitope specific antibodies. In addition, comparison of titers from synthetic peptide and recombinant protein investigations could provide more information on humoral immune response properties.

Even though the predominance of peptide specific antibody presence in AIT patients is truly intriguing, the chronic inflammation present in these patients, may have influenced the production of HHV-6 antigen specific antibodies by polyclonal B cells. Insights into the true clinical significance of these viral antibodies and their emergence (whether by cell surface viral proteins or polyclonal B cells) may be further gathered by analyzing samples from patients without HHV-6 in their thyroid gland (28).

Although, antibodies specific for synthetic peptides did not cross-react with human recombinant proteins, the possibility of cross-reactive auto-antibodies specific to conformational epitopes still may exist as human chemokine receptor CCR1, CCR3, and CCR5 specific IgG and IgM antibodies were found in AIT patients’ and blood donors’ plasma. To further elucidate the potential role of these viral chemokine receptors as autoimmunity triggers, conformational epitopes, antibodies specific to them and their cross-reactive abilities should be analyzed.

Despite the lack of peptide antibody cross-reactivity, we have demonstrated that the two proteins of HHV-6—U12 and U51 are immunogenic, with multiple immunogenic epitopes. We have revealed clear differences in the viral peptide immune response patterns in AIT patients vs donors, with patients having predominantly peptide specific IgGs, while donors—IgMs. Moreover, IgG response against HHV-6 U12 and U51 derived peptides was shown to be associated with virus load and was stronger in patients with high anti-thyroid auto-antibody levels. Since we and others have demonstrated the high prevalence of HHV-6 in diseased thyroid glands, information of an immunogenic viral antigen expressed in the tissues and the other results acquired in this study raise questions and provide new insights and new venues for research on how HHV-6 could exacerbate the autoimmune process.

## MATERIALS AND METHODS

### Proteins and peptides.

Viral GPCR homologues were aligned with human CCR1, CCR3, and CCR5 based on their sequence and functional similarity to HHV-6 U12 and U51. Alignment of viral GPCR homologues (HHV-6A/B U12 and U51) with human chemokine receptors (CCR1, CCR3, and CCR5) was done by T-coffee software (http://tcoffee.crg.cat/; version_11.00.d625267). Simultaneously, Bepipred Linear Epitope Prediction and Kolaskar & Tongaonkar algorithms (http://tools.iedb.org/bcell/) were used for linear epitope prediction. Data from the amino acid alignment was combined with linear epitope predictions in order to design 20 mer synthetic peptides (Figs. S1–S2 in the supplemental material). All synthetic peptides were modified with three polyethylene glycol (PEG3) molecules at amino terminus end. PEG3 was used as a spacer in carbodiimide coupling of peptides to carboxylated magnetic beads, to provide better freedom to present epitopes to antibodies during SMIA protocol.

HHV-6 U12 and U51 amino-acids sequences from both HHV-6 variants A and B, were used in the peptide design. Peptides with identical amino acid sequences in both HHV-6 species proteins as well as peptides with some amino acid differences corresponding to each of the species proteins were designed. In total 28 synthetic peptides were designed. Sequences, source and positioning of the synthetic peptides evaluated in this study are given in Tables S1–S2 of the supplementary materials.

The 20 mer peptides with PEG3 modification at amino terminus end were synthesized by GenScript (USA). Lyophilized peptides were dissolved in sterile phosphate-buffered saline (PBS), pH 7. The dissolution of peptides sometimes had to be facilitated by warming of the solution to 37°C overnight on a shaker or by sonication for 5 min. Highly hydrophobic peptides were dissolved in 10% to 50% (vol/vol) dimethyl sulfoxide (DMSO) (Sigma D2650).

Human recombinant CCR1, CCR3, and CCR5 were obtained from Abnova (Taipei, Taiwan). The proteins were produced *in vitro* using wheat germ expression system which preserves native protein folding.

### Study group.

Plasma samples of 79 AIT patients (9 males [11%], 70 females [89%], median age of 48 [inter-quartile range [IQR]: 39-60]) whose thyroid tissue samples were acquired after thyroidectomy and were shown to be positive for HHV-6 genomic sequences were enrolled in this study. No indications of inherited chromosomally integrated HHV-6 cases were present in the patient group, as whole blood HHV-6 positivity was rarely observed and if observed, harbored extremely low viral loads (7, 16). In all cases, indication for surgery was nodular goiter. Plasma samples from 32 blood donors (BD) (5 males [16%], 27 females [84%], median age of 39 [inter-quartile range [IQR]: 27-48]), which were negative on anti-TPO (thyroid peroxidase), anti-TG (thyroglobulin) and anti-TSHr (thyroid stimulating hormone receptor) antibodies, were enrolled in the study as a control group. HHV-6B was detected in all patients’ samples enrolled in this study. All tissue samples were received from the Riga East Clinical University Hospital.

The permission to conduct the research was received from the Rīga Stradiņš University (RSU) Ethics Committee (Nr. 69/22.02.2018) and all participants included in the study gave their written consent prior to the examinations.

### Coupling of the synthetic peptides and human recombinant proteins to magnetic carboxylated microspheres.

The synthetic peptides and human recombinant proteins were coupled to Luminex Magnetic Carboxylated Microspheres (MagPlex) using xMAP Antibody Coupling Kit (Millipore, USA), according to manufacturer protocol. Use of Luminex technology allows to run one immune suspension reaction using various different antigens simultaneously. Beads with 100 different regions—each region bearing specific (self-recognition) dye mark, could be used in a single reaction. Therefore, it is possible to distinguish multiple immunogenic signals from different antigen reactions. The final pellet was re-suspended in an appropriate amount of StabilGuard buffer (200 μL of StabilGuard buffer if 100 μL of beads were taken). This created a bead mixture consisting of 1,250 beads/μL. The coupled beads were stored at +4°C in the dark.

### SMIA for the detection of IgG and IgM antibodies specific to synthetic peptides and human recombinant proteins.

Antigens were coupled covalently to carboxylated color-coded beads as described above. Also, in each reaction naked beads were included to exclude nonspecific signals caused by nonspecific binding to the beads. IgG was detected using biotinylated protein G (BPG) as a secondary antibody (Sigma-Aldrich, cat. Nr. P8045). For IgM antibody detection biotinylated anti-human IgM (affinity purified, μ-chain specific, Sigma-Aldrich cat. Nr. B1140) was used as a secondary antibody. To exclude IgG and rheumatoid factor (Rf) nonspecific binding during IgM run, IgG/Rf Stripper (Bio-Rad, USA) was used for all samples according to manufacturer protocol.

The assay system requires only 5 μL of plasma. Plasma samples were diluted in the ratio of 1:10 with StabilGuard buffer (Surmodics, USA). It is an immunoassay stabilizer (BSA-Free) which effectively preserves the conformation and activity of dried proteins while simultaneously blocking the surface to reduce nonspecific binding. Also used as an antibody stabilizer.

All reagents and samples were adjusted to room temperature before beginning the assay. Coupled beads were vortexed 10 sec and sonicated 10 sec before adding to the bead mixture.

The assay starts with the incubation of plasma samples with the magnetic bead mixture. 50 μL of the diluted plasma sample was added to the appropriate wells, 50 μL of PBS was added to the well designated as the blank value, and 50 μL of StabilGuard was added to the well designated as the none antigen control (NAC). 50 μL of bead mixture (containing 100 beads/μL of each antigen coupled beads’ region) was added to all wells and the plate was incubated with agitation at room temperature in the dark for 1 h. After the incubation the plate was placed on the magnet for 60 sec and aspirated, followed by 3 washes with 200 μL PBS.

Initially beads were re-suspended in 50 μL SatbilGuard and 50 μL of secondary antibodies were added. However, our first results showed relatively high NAC signals. This could be explained by the fact that the human blood samples contained enough non-antibody protein (a typical serum or plasma contains 70 mg/mL) to block the low affinity detected by the NAC. The StabilGuard diluent does not contain protein. We did not routinely include highly concentrated blocking protein in the tests because of occasional clogging of the probe in the Luminex machine in the presence of blocking protein. This was was observed very often when we were using non-magnetic beads and filter plates. Since in this study we used xMAP magnetic beads and standard solid plates we decided to change StabilGuard to 500 μg/mL ovalbumin (OVA) in PBS. Such buffer was used according to published supplementary data and was effective in lowering median fluorescence intensity (MFI) values of NAC (17). Prior to addition, the secondary antibodies (biotinylated protein G or biotinylated anti-human IgM) were diluted in SatbilGuard to the concentration 4 μg/mL. The plate was incubated with agitation at room temperature in the dark for 30 min. After incubation the plate was washed as described above.

Beads were again re-suspended in 50 μL OVA buffer, followed by the addition of 50 μL of streptavidin-phycoerythrin diluted in StabilGuard to the concentration 4 μg/mL. The plate was incubated with agitation at room temperature in the dark for 15 min. After incubation the plate was washed twice, and the beads re-suspended in 50 μL of StabilGuard. Plate was read using Luminex 200 system, which beforehand was calibrated using Luminex 200 Calibration Kit.

As 77% of humans are exposed to HHV-6B by the age of two and the prevalence of HHV-6 in adults is assumed to be over 90%, it is almost impossible to find HHV-6 negative individuals for the control group for the evaluation of a cut off. To distinguish positive IgG and IgM signals from nonspecific signals, a run with preabsorbed samples with each soluble peptide (50 ng/50 μL of diluted plasma) samples was done. Samples were incubated with the peptides for an hour in room temperature with agitation prior to magnetic bead addition. The results were reported as percentage of MFI reduction after pre-absorbing with peptides calculated according to the following formula: [(MFI non-preabsorbed–MFI preabsorbed)/MFI non-preabsorbed] × 100. A reduction equal or greater than 35% was used to discriminate IgG/IgM-positive from IgG/IgM-negative samples (18).

The same protocol with pre-absorption was followed to test possible synthetic peptides antibody cross-reactivity with human recombinant CCRs. Pre-absorption was done with 25 ng of each CCR (CCR1, CCR3, and CCR5) in 50 μL AIT and BD plasma pools, as well as separate individuals’ plasma which were highly positive on synthetic peptides immune response. Non-preabsorbed samples were run simultaneously with preabsorbed in SMIA and MFI signals were compared.

### Statistical analysis of the data.

Data analysis and visualization was done using the GraphPad Prism software (USA), 8.4.3 version. Following statistical tests were utilized—Mann-Whitney U test and Chi-square tests.

### Data availability.

All data generated or analyzed during this study is included in the main text and supplementary material, including amino acid sequences of proteins and acquired synthetic peptides in FASTA fromat (Figs. S1–S2 and Tables S1–S2 in the supplemental material).

Source - UniProt database (https://www.uniprot.org/; all accession numbers included in Supplementary Information). Sequences were aligned using T-COFFEE software (http://tcoffee.crg.cat/; version_11.00.d625267). Two linear epitope prediction algorithms were Bepipred Linear Epitope Prediction and Kolaskar & Tongaonkar on IEDB Analysis Resource website (http://tools.iedb.org/bcell/). All relevant data are also available upon request from the corresponding author.
