# Supplementary material for: Investigation of the Involvement of HHV-6 Encoded Viral Chemokine Receptors in Autoimmune Thyroiditis Development
Source: Microbiol Spectr. 2022 May 23;10(3):e02369-21. doi: 10.1128/spectrum.02369-21 (PMC9241611; doi:10.1128/spectrum.02369-21)
Supplement: SUPPLEMENTAL FILE 1 — Fig. S1 to S10 and Tables S1 and S2. Download spectrum.02369-21-s001.pdf, PDF file, 0.8 MB [file spectrum.02369-21-s001.pdf]

Supplementary information

1. Protein amino acid sequences used in this study (FASTA). Source – UniProt database

>HHV6A_U12_APO39151.1
MDTVIELSKLLRNEEFKGNDSCTSTPTLKTARIMESAVTGITLTTSIPMIIIVVTTMILYHRVAKHNATS
FYVITLFASDFVLMWCVFFMTVNRKQLFSFNRFFCQLVYFIYHAVCSYSISMLAIIATIRYKTLHRRKKT
ESKTSSTGRNIGILLLASSMCAIPTALFVKTNGMKKTGKCVVYISSKKAYELFLAVKIVFSFIWGVLPTM
VFSFFYVIFCKALHDVTEKKYKKTLFFIRILLLSFLLIQIPYIAILICEIAFLYMPQNTCFWLARVEILQ
LIIRLMPQVHCFSNPLVYAFTGGELRNRFTACFQSFFPKTLCSTQKRNGSDVSEHDQNSKSKASVEKNQP
L

>HHV6B_U12_APO38312.1
MDTVIELSKLLHDEEFKDNASCTSTPTLKTARIIESAVTGITLTASVPMIIIVITTMILYHRVAKHNATS
FYVITLFASDFVLMWCVFFMTVNREQLFSFNRFFCQLVYFIYHAVCSYSISMLAIIATIRYKTLHRRKQT
ESKTYSTGRNIGILLLASSMCAIPTALFVQINGAKKTTGKCVVYLSSPKAYELFLAVKIVFSFIWGVLPT
MVFSFFYFIFCKALHGVTKKKHKKTLFFISILLLSFLLIQIPYIAILISEIAFLYMPQNTCFWLARAEIL
QLIIRLMPQVHCFSNPLVYAFTGGELRNRFTTCFQCPFFPKRLCSTQNRKQSDVSEHDQNSPSESSVDEN
EPP

>HHV6A_U51_APO39190.1
MGKETKSLAWPATAEFYGWVFIFSSIQLCTMVLLTVRFNSFKVGREYAVFTFAGMSFNCFLLPIKMGLLS
GHWSLPRDFCAILLYIDDFSIYFSSWSLVFMAIERINHFCYSTPLLNENSKALAKVCFPIVWIISGVQAL
QMLNNYKATALQNETPQCFLAFLRSGHDMWLMLVYSVMIPVMLVFIYLYSKNFMLLKDELSTVTTYLCIY
LLLGTIAHLPKAGLSEIESDKIFYGLRDIFMALPVLKVYYIPVMAYCMACDDHTVPVRLCSIWLVNLCKK
CFSCTRREKESDLEVGIKMLK

>HHV6B_U51_APO38351.1
MEKETKSLAWPATAEFYGWVFIFSSIQLCTVVFLTVRFNGFKVGREYAVFTFAGMSFNCFLLPIKMGLLS
GHWTLPRDFCAILLYIDDFSAYFSSWSLVFMAIERINYFCYSTPLLNENSKALAKVCFPIVWVVSGVQAL
QMLNNYKATALQNETGQCFLAFLRSGHDMWLMLVYSVVIPVMLVFFYLYSKNFMLLKDELSSVTTYLCIY
LLLGTIAHLPKAALSEIESDKIFYGLRDIFMALPVLKVYYISAMAYCMACDDHTVPVRLCSIWLVNLCKK
CFSCTRREKGSDLEVGIKMLK

>Human_CCR1_NP_001286.1
METPNTTEDYDTTTEFDYGDATPCQKVNERAFGAQLLPPLYSLVFVIGLVGNILVVLVLVQYKRLKNMTS
IYLLNLAISDLLFLFTLPFWIDYKLKDDWVFGDAMCKILSGFYYTGLYSEIFFIILLTIDRYLAIVHAVF
ALRARTVTFGVITSIIIWALAILASMPGLYFSKTQWEFTHHTCSLHFPHESLREWKLFQALKLNLFGLVL
PLLVMIICYTGIIKILLRRPNEKKSKAVRLIFVIMIIFFLFWTPYNLTILISVFQDFLFTHECEQSRHLD
LAVQVTEVIAYTHCCVNPVIYAFVGERFRKYLRQLFHRRVAVHLVKWLPFLSVDRLERVSSTSPSTGEHE
LSAGF

>Human_CCR3_AAL85154.1
MTTSLDTVETFGTTSYYDDVGLLCEKADTRALMAQFVPPLYSLVFTVGLLGNVVVVMILIKYRRLRIMTN
IYLLNLAISDLLFLVTLPFWIHYVRGHNWVFGHGMCKLLSGFYHTGLYSEIFFIILLTIDRYLAIVHAVF
ALRARTVTFGVITSIVTWGLAVLAALPEFIFYETEELFEETLCSALYPEDTVYSWRHFHTLRMTIFCLVL
PLLVMAICYTGIIKTLLRCPSKKKYKAIRLIFVIMAVFFIFWTPYNVAILLSSYQSILFGNDCERSKHLD
LVMLVTEVIAYSHCCMNPVIYAFVGERFRKYLRHFFHRHLLMHLGRYIPFLPSEKLERTSSVSPSTAEPE
LSIVF

>Human_CCR5_AAB57793.1
MDYQVSSPIYDINYYTSEPCQKINVKQIAARLLPPLYSLVFIFGFVGNMLVILILINCKRLKSMTDIYLL
NLAISDLFFLLTVPFWAHYAAAQWDFGNTMCQLLTGLYFIGFFSGIFFIILLTIDRYLAVVHAVFALKAR
TVTFGVVTSVITWVVAVFASLPGIIFTRSQKEGLHYTCSSHFPYSQYQFWKNFQTLKIVILGLVLPLLVM
VICYSGILKTLLRCRNEKKRHRAVRLIFTIMIVYFLFWAPYNIVLLLNTFQEFFGLNNCSSSNRLDQAMQ
VTETLGMTHCCINPIIYAFVGEKFRNYLLVFFQKHIAKRFCKCCSIFQQEAPERASSVYTRSTGEQEISV
GL

2. Alignment of viral and human chemokine receptor amino acid sequences and linear epitope prediction

Sequences were aligned using T-COFFEE software. Two linear epitope prediction algorithms were used - Bepipred Linear Epitope Prediction (BLEP) and Kolaskar & Tongaonkar (K&T).

2.1. HHV-6 U12 (A and B)

```
T-COFFEE, Version_11.00.d625267 (2016-01-11 15:25:41 - Revision d625267 - Build 507)
Cedric Notredame
CPU TIME:0 sec.
SCORE=789
*
 BAD AVG GOOD
*
HHV6A_U12_APO39    :  76
HHV6B_U12_APO38    :  75
Human_CCR1_NP_0    :  77
Human_CCR3_AAL8    :  77
Human_CCR5_AAB5    :  77
cons               :  78

HHV6A_U12_APO39    MDTVIELSKLLRNEEFKG--NDSCTSTPTLKT-ARIMESAVTGITLTTSIPMIIIVVTTMILY
HHV6B_U12_APO38    MDTVIELSKLLHDEEFKD--NASCTSTPTLKT-ARIIESAVTGITLTASVPMIIIVITTMILY
Human_CCR1_NP_0    METPNTTEDYDTTTEFDYGDATPCQKVNERAFGAQLLPP-LYSLVFVIGLVGNILVVLVLVQY
Human_CCR3_AAL8    MTTSLDTVETFGTTSYYDDVGLLCEKADTRALMAQFVPP-LYSLVFTVGLLGNVVVVMILIKY
Human_CCR5_AAB5    MDYQVSS--PIYDINYYT--SEPCQKINVKQIAARLLPP-LYSLVFIFGFVGNMLVILILINC

cons               *             .:       * .       *::: . : .:.:  ..   ::*:  ::


HHV6A_U12_APO39    HRVAKHNATSFYVITLFASDFVLMWCV-FFMTVNRKQLFSFNRFFCQLVYFIYHAVCSYSISM
HHV6B_U12_APO38    HRVAKHNATSFYVITLFASDFVLMWCV-FFMTVNREQLFSFNRFFCQLVYFIYHAVCSYSISM
Human_CCR1_NP_0    KRL--KNMTSIYLLNLAISDLLFLFTLPFWIDYKLKDDWVFGDAMCKILSGFYYTGLYSEIFF
Human_CCR3_AAL8    RRL--RIMTNIYLLNLAISDLLFLVTLPFWIHYVRGHNWVFGHGMCKLLSGFYHTGLYSEIFF
Human_CCR5_AAB5    KRL--KSMTDIYLLNLAISDLFFLLTVPFWAHY-AAAQWDFGNTMCQLLTGLYFIGFFSGIFF

cons               :*:  :  *.:*::.*  **:.::  : *:        : *.  :*:::  :*.      * :
```

```
HHV6A_U12_APO39    LAIIATIRYKT-LHRRKKTESKTSSTGRNIGILLLAS-SMCAIPTALFVKTNGMKK-TGKCVV
HHV6B_U12_APO38    LAIIATIRYKT-LHRRKQTESKTYSTGRNIGILLLAS-SMCAIPTALFVQINGAKKTTGKCVV
Human_CCR1_NP_0    IILLTIDRYLAIVHAVFALRARTVTFGVITSIIIWALAILASMPGLYFSKTQWEFT-HHTCSL
Human_CCR3_AAL8    IILLTIDRYLAIVHAVFALRARTVTFGVITSIVTWGLAVLAALPEFIFYETEELFE-ETLCSA
Human_CCR5_AAB5    IILLTIDRYLAVVHAVFALKARTVTFGVVTSVITWVVAVFASLPGIIFTRSQKEGL-HYTCSS

cons               : :::  ** : :*       .::* : *  .::        :.::*   * . :        *

HHV6A_U12_APO39    YI--SSKKAYELFLAVKIVFSFIWGVLPTMVFSFFYVIFCKALHDVTE-KKYKKTLFFIRILL
HHV6B_U12_APO38    YL--SSPKAYELFLAVKIVFSFIWGVLPTMVFSFFYFIFCKALHGVTK-KKHKKTLFFISILL
Human_CCR1_NP_0    HFPHESLREWKLFQALKLNL--FGLVLPLLVMIICYTGIIKILLRRPN-EKKSKAVRLIFVIM
Human_CCR3_AAL8    LYPEDTVYSWRHFHTLRMTI--FCLVLPLLVMAICYTGIIKTLLRCPS-KKKYKAIRLIFVIM
Human_CCR5_AAB5    HFPYSQYQFWKNFQTLKIVI--LGLVLPLLVMVICYSGILKTLLRCRNEKKRHRAVRLIFTIM

cons                  .    :. * :::: :  :  *** :*: : *  : * *     . :*  ::: :*  ::

HHV6A_U12_APO39    LSFLLIQIPYIAILICEIAFLYMPQNTCFWLARVEILQLIIRLMPQVHCFSNPLVYAFTGGEL
HHV6B_U12_APO38    LSFLLIQIPYIAILISEIAFLYMPQNTCFWLARAEILQLIIRLMPQVHCFSNPLVYAFTGGEL
Human_CCR1_NP_0    IIFFLFWTPYNLTILISVFQDFLFTHECEQSRHLDLAVQVTEVIAYTHCCVNPVIYAFVGERF
Human_CCR3_AAL8    AVFFIFWTPYNVAILLSSYQSILFGNDCERSKHLDLVMLVTEVIAYSHCCMNPVIYAFVGERF
Human_CCR5_AAB5    IVYFLFWAPYNIVLLLNTFQEFFGLNNCSSSNRLDQAMQVTETLGMTHCCINPIIYAFVGEKF

cons                                                                 **::***.*  .:

HHV6A_U12_APO39    RNRFTACFQS-FFPKTLCSTQKRNGSDVSEHDQNSKSKASVEKNQ---PL
HHV6B_U12_APO38    RNRFTTCFQCPFFPKRLCSTQNRKQSDVSEHDQNSPSESSVDENE---PP
Human_CCR1_NP_0    RKYLRQLFHR-RVAVHLVKWLPFLSVDRLERVSST-SPS-TGEHELSAGF
Human_CCR3_AAL8    RKYLRHFFHR-HLLMHLGRYIPFLPSEKLERTSSV-SPS-TAEPELSIVF
Human_CCR5_AAB5    RNYLLVFFQK-HIAKRFCKCCSIFQQEAPERASSVYTRS-TGEQEISVGL

cons               *: :   *:  .   :         :  *: ..  : : . : :
```

Supplementary figure 1 (continued from page 2). Alignment of HHV-6A/B U12 protein with human CCR1, 3 and 5 using T-COFFEE software. Highly conservative regions are shown in pink. Predicted linear epitopes - BLEP [underlined] and K&T [strikethrough]

Supplementary table 1. Peptides designed from HHV6A/B U12 proteins' amino acid sequences with their source and positioning as well as predicted linear epitopes. Linear epitopes predicted with BLEP algorithm are underlined and epitopes predicted with K&T are highlighted by strikethroughs. All peptides were 20-mers and had a three-carbon polyethylene glycol spacer coupled at the amino end. The spacer started with a primary amino group.

Sequences' source and positioning	Peptides' identification	Sequence and predicted linear epitope (BLEP [underlined], K&T [strikethrough])
HHV6A_U12_APO39151.1 (11-30)	HHV6_GPCR1	LRNEEFKGNDSCTSTPTLKT
HHV6A_U12_APO39151.1 (42 -61)	HHV6_GPCR2	TLTTSIP~~MIIIVVTTM~~ILYH
HHV6A_U12_APO39151.1 (130-149)	HHV6_GPCR3	RYKTLHRRKKTESKTSSTGR
HHV6A_U12_APO39151.1 (141-160)	HHV6_GPCR4	ESKTSSTGRNIG~~ILLLASSM~~
HHV6A_U12_APO39151.1 (189-208) HHV6B_U12_APO38312.1 (190-209)	HHV6_GPCR5	AY~~ELFLAVKIVFSFIWGV~~LP
HHV6A_U12_APO39151.1 (212-231)	HHV6_GPCR6	F~~SFFYVIFCKALHD~~VTEKKY
HHV6A_U12_APO39151.1 (280-299) HHV6B_U12_APO38312.1 (281-300)	HHV6_GPCR7	~~QLIIRLMPQVHCFSNPLVYA~~
HHV6B_U12_APO38312.1 (11-30)	HHV6_GPCR8	LHDEEFKDNASCTSTPTLKT
HHV6B_U12_APO38312.1 (42-61)	HHV6_GPCR9	TLTASV~~PMIIIVITT~~MILYH
HHV6A_U12_APO39151.1 (71-90) HHV6B_U12_APO38312.1 (71-90)	HHV6_GPCR10	~~FYVITL~~FASDFV~~LMWCVFFM~~
HHV6A_U12_APO39151.1 (102-121) HHV6B_U12_APO38312.1 (102-121)	HHV6_GPCR11	RF~~FCQLVYFIYHAVCSYSIS~~
HHV6B_U12_APO38312.1 (141-160)	HHV6_GPCR12	ESKTYSTGRNIG~~ILLLASSM~~
HHV6B_U12_APO38312.1 (162-181)	HHV6_GPCR13	A~~IPTALFVQ~~INGAKKTTGKC
HHV6B_U12_APO38312.1 (184-203)	HHV6_GPCR14	~~YLSSP~~KAY~~ELFLAVKIVFSF~~
HHV6B_U12_APO38312.1 (213-232)	HHV6_GPCR15	F~~SFFYFIFCKALHG~~VTKKKH
HHV6B_U12_APO38312.1 (302-321)	HHV6_GPCR16	TGGELRNRFTT~~CFQCPFF~~PK

2.2. HHV-6 U51 (A and B)

```
T-COFFEE, Version_11.00.d625267 (2016-01-11 15:25:41 - Revision d625267 - Build 507)
Cedric Notredame
CPU TIME:0 sec.
SCORE=847
*
 BAD AVG GOOD
*
HHV6A_U51_APO39  :  87
HHV6B_U51_APO38  :  87
Human_CCR1_NP_0  :  86
Human_CCR3_AAL8  :  87
Human_CCR5_AAB5  :  87
cons             :  84

HHV6A_U51_APO39  MGKE-------------------------TKSLAWPATAEFYGWVFIFSSIQLCTMVLLTVRF
HHV6B_U51_APO38  MEKE-------------------------TKSLAWPATAEFYGWVFIFSSIQLCTVVFLTVRF
Human_CCR1_NP_0  METPNTTED-YDTTTEFDYGDATPCQKVNERAFGAQLLPPLYSLVFVIGLVGNILVVLVLVQY
Human_CCR3_AAL8  MTTSLDTVETFGTTSYYD-DVGLLCEKADTRALMAQFVPPLYSLVFTVGLLGNVVVVMILIKY
Human_CCR5_AAB5  MDYQVSSPI-YDIN-Y---YTSEPCQKINVKQIAARLLPPLYSLVFIFGFVGNMLVILILINC

cons             *                             : :     . :*. ** .. :    :::: :.

HHV6A_U51_APO39  NSFKVGREYAVFTFAGMS-FNCFLLPIKMGLLS-GHWSLPRDFCAILLYIDDFSIYFSSWSLV
HHV6B_U51_APO38  NGFKVGREYAVFTFAGMS-FNCFLLPIKMGLLS-GHWTLPRDFCAILLYIDDFSAYFSSWSLV
Human_CCR1_NP_0  KRLKNMTSIYLLNLAISDLLFLFTLPFWIDYKLKDDWVFGDAMCKILSGFYYTGLYSEIFFII
Human_CCR3_AAL8  RRLRIMTNIYLLNLAISDLLFLVTLPFWIHYVRGHNWVFGHGMCKLLSGFYHTGLYSEIFFII
Human_CCR5_AAB5  KRLKSMTDIYLLNLAISDLFFLLTVPFWAHYAA-AQWDFGNTMCQLLTGLYFIGFFSGIFFII

HHV6A_U51_APO39  FMAIERINHFCYSTPLLNENSKALAKVCFPIVWIISGVQALQMLNNYKATALQNETPQCFLAF
HHV6B_U51_APO38  FMAIERINYFCYSTPLLNENSKALAKVCFPIVWVVSGVQALQMLNNYKATALQNETGQCFLAF
Human_CCR1_NP_0  LLTIDRYLAIVHAVFALRARTVTFGVITSIIIWALAILASMPGLYFSKTQW-EFTHHTCSLHF
Human_CCR3_AAL8  LLTIDRYLAIVHAVFALRARTVTFGVITSIVTWGLAVLAALPEFIFYETEE-LFEETLCSALY
Human_CCR5_AAB5  LLTIDRYLAVVHAVFALKARTVTFGVVTSVITWVVAVFASLPGIIFTRSQK-EGLHYTCSSHF

cons             :::*:*   . ::.  *. .: ::. :   : * :: . ::  :   .:         *   :
```

```
HHV6A_U51_APO39   LRSGHDMWLM------LVYSVMIPVMLVFIYLYSKNFMLLKDELSTVTTYLCIYLLLGTIAHL
HHV6B_U51_APO38   LRSGHDMWLM------LVYSVVIPVMLVFFYLYSKNFMLLKDELSSVTTYLCIYLLLGTIAHL
Human_CCR1_NP_0   PHESLREWKLFQALKLNLFGLVLPLLV-MIICYTG--------------------IIKIL---
Human_CCR3_AAL8   PEDTVYSWRHFHTLRMTIFCLVLPLLV-MAICYTG--------------------IIKTL---
Human_CCR5_AAB5   PYSQYQFWKNFQTLKIVILGLVLPLLV-MVICYSG--------------------ILKTL---

cons                .    *         :  :::*::: :   *:                     ::  :


HHV6A_U51_APO39   PKAGLSEIES-DKIFYGLRDIFMALPVLKVYYIP-----------------------------
HHV6B_U51_APO38   PKAALSEIES-DKIFYGLRDIFMALPVLKVYYIS-----------------------------
Human_CCR1_NP_0   -----LRRPN-EKKSKAVRLIFVIMIIFFLFWTPYNLTILISVFQDFLFTHECEQSRHLDLAV
Human_CCR3_AAL8   -----LRCPS-KKKYKAIRLIFVIMAVFFIFWTPYNVAILLSSYQSILFGNDCERSKHLDLVM
Human_CCR5_AAB5   -----LRCRNEKKRHRAVRLIFTIMIVYFLFWAPYNIVLLLNTFQEFFGLNNCSSSNRLDQAM

cons                          *    .* **  : :  ::: .

HHV6A_U51_APO39   ----VMAYCMACDDHTVP-----------------------VRLCSIWLVNLCKKCFSC-TRR
HHV6B_U51_APO38   ----AMAYCMACDDHTVP-----------------------VRLCSIWLVNLCKKCFSC-TRR
Human_CCR1_NP_0   QVTEVIAYTHCCVNPVIYAFVGERFRKYLRQLFHRRVAVHLVKWLPFLSVDRLERVSST-SPS
Human_CCR3_AAL8   LVTEVIAYSHCCMNPVIYAFVGERFRKYLRHFFHRHLLMHLGRYIPFLPSEKLERTSSV-SPS
Human_CCR5_AAB5   QVTETLGMTHCCINPIIYAFVGEKFRNYLLVFFQKHIAKRFCKCCSIFQQEAPERASSVYTRS

cons                  .:.   .* :  :                         :  .:   :  ::  *  :


HHV6A_U51_APO39   EKESDLEVGIKMLK
HHV6B_U51_APO38   EKGSDLEVGIKMLK
Human_CCR1_NP_0   TGEHELSAGF----
Human_CCR3_AAL8   TAEPELSIVF----
Human_CCR5_AAB5   TGEQEISVGL----

cons                  ::.  :
```

Supplementary figure 2 (continued from page 5). Alignment of HHV-6A/B U12 protein with human CCR1, 3 and 5 using T-COFFEE software. Highly conservative regions are shown in pink. Predicted linear epitopes - BLEP [underlined] and K&T [strikethrough]

Supplementary table 2. Peptides designed from HHV6A/B U51 proteins' amino acid sequences with their source and positioning as well as predicted linear epitopes. Linear epitopes predicted with BLEP algorithm are underlined and epitopes predicted with K&T are highlighted by strikethroughs. All peptides were 20-mers and had a three-carbon polyethylene glycol spacer coupled at the amino end. The spacer started with a primary amino group.

Sequence origin and positioning	Peptides' sequence short name	Sequence (Bepipred Linear Epitope Prediction [underlined] and Kolaskar & Tongaonkar [strikethroughs])
HHV6A_U51_APO39190.1 (1-20)	GPCR17	<u>MGKETKSLAWP</u>ATAEFYGWV
HHV6A_U51_APO39190.1 (18-37)	GPCR18	G~~WVFIFSSIQLCTMVLLTV~~R
HHV6A_U51_APO39190.1 (108-127)	GPCR19	HFCYSTPL<u>LNE</u>NSKA~~LAKVC~~
HHV6A_U51_APO39190.1 (146-165)	GPCR20	YKAT<u>ALQNET</u>P~~QCFLAF~~LRS
HHV6A_U51_APO39190.1 (261-280) HHV6B_U51_APO38351.1 (261-280)	GPCR21	D~~DHTVPVRLCSIWLVNLCKK~~
HHV6B_U51_APO38351.1 (1-20)	GPCR22	<u>MEKETKSLAWP</u>ATAEFYGWV
HHV6B_U51_APO38351.1 (18-37)	GPCR23	GWVF~~IFSSIQLCTVVFLT~~VR
HHV6B_U51_APO38351.1 (73-92)	GPCR24	WTLPR~~DFCAILLYI~~DDFSAY
HHV6B_U51_APO38351.1 (108-127)	GPCR25	~~YFCYST~~PL<u>LNE</u>NSKA~~LAKVC~~
HHV6B_U51_APO38351.1 (170-189)	GPCR26	WL~~MLVYSVVIPVMLVFFYLY~~
HHV6B_U51_APO38351.1 (197-216)	GPCR27	DELSS~~VTTYLCIYLLLGTI~~
HHV6B_U51_APO38351.1 (218-237)	GPCR28	HLPKAAL<u>SEI</u>ESDKIFYGLR

3. Figures not included in the main text

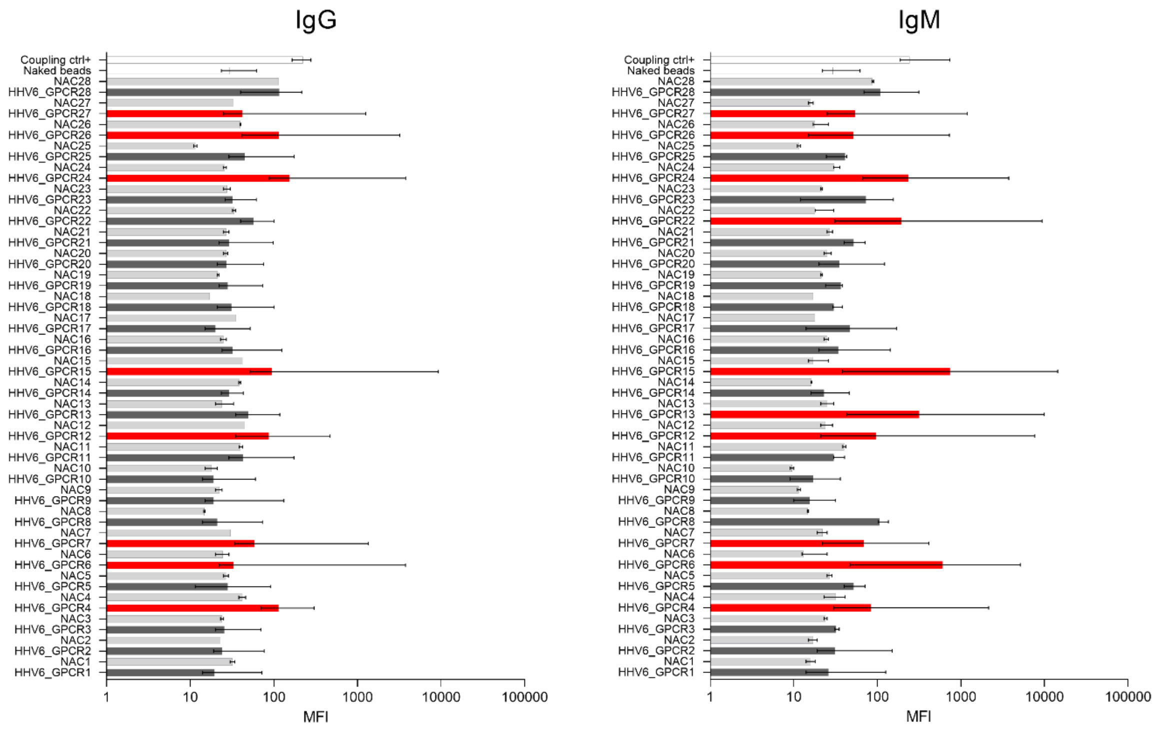

Supplementary figure 3. SMIA results of 28 synthetic peptides immunogenicity showing median fluorescence intensity (MFI) values for IgG and IgM class antibodies. Peptides highlighted in red represent the most immunogenic ones, which were used for further investigations. Lighter grey bars represent none antigen controls (NAC) of each peptide.

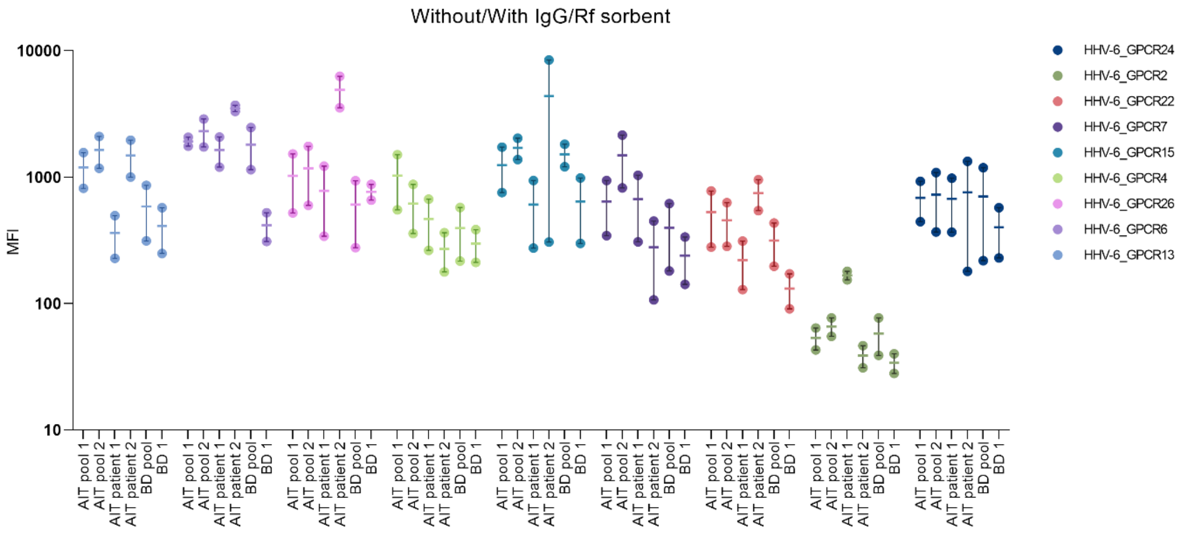

Supplementary figure 4. Comparison of IgM levels without and with IgG and Rf sorbent. Higher dots plotted represent the values of samples not treated with the sorbent. Measurements were done in two autoimmune thyroiditis (AIT) patient pooled samples and an individual AIT patient sample, which had previously shown elevated levels of peptide antibodies. Additionally, measurements were done in one blood donor pooled sample and an individual blood donor sample.

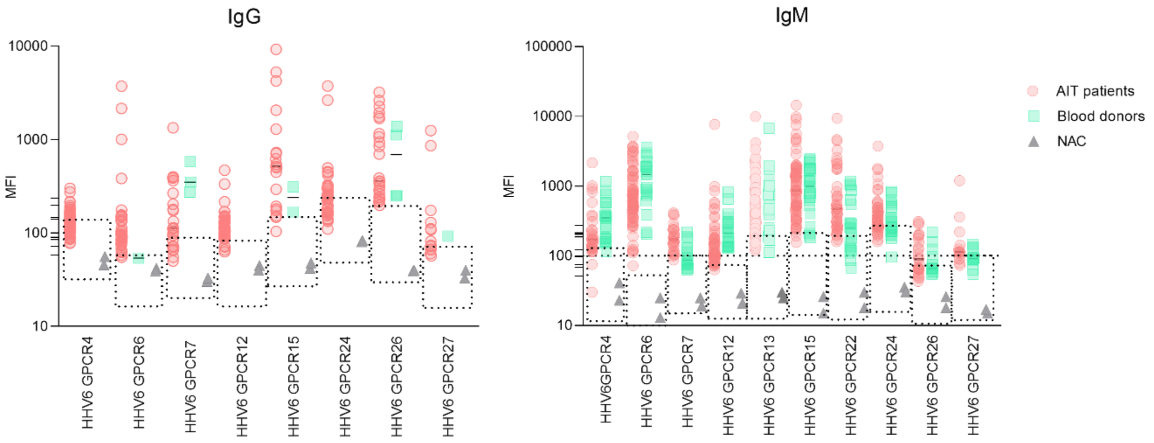

Supplementary figure 5. SMIA results of immunogenic peptides immunogenicity showing MFI values for IgG and IgM class antibodies in AIT patients', and blood donors' plasma samples which showed >35% change after preabsorption treatment with the same peptides (samples were run in parallel – without and with preabsorption). With punctuation line showing additional cut off for each peptide signal using mean value of negatives with 2 SD.

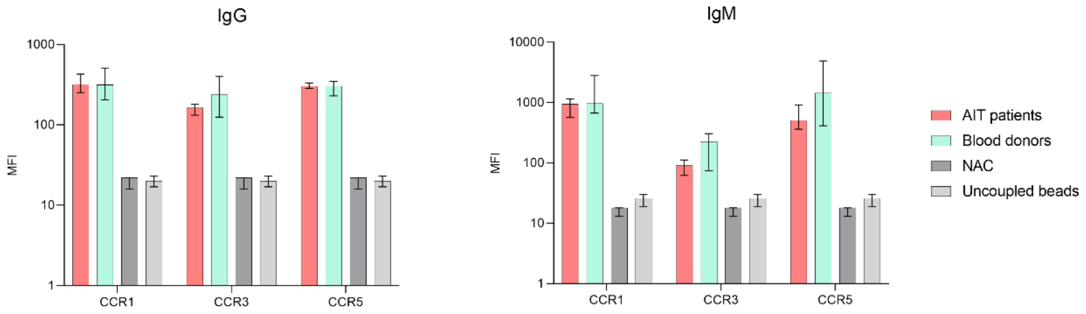

Supplementary figure 6. IgG and IgM specific antibodies to human recombinant proteins (CCR1, CCR3 and CCR5) detected by SMIA in AIT patients' and blood donors plasma pools.

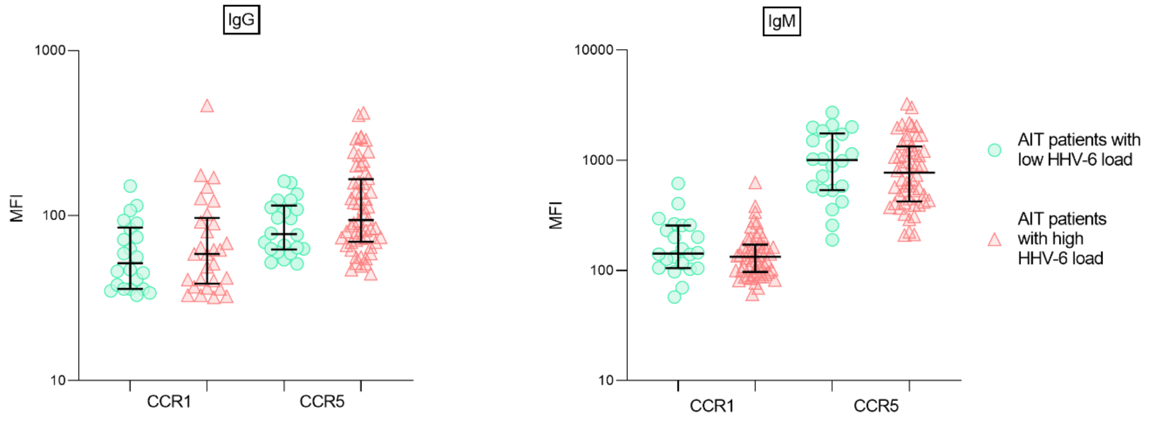

Supplementary figure 7. Comparison of antibodies levels against human CCR1 and CCR5 (the most reactive proteins) between AIT patients with low and high HHV-6 load (<200 and >200 copies/10^6 cells, respectively).

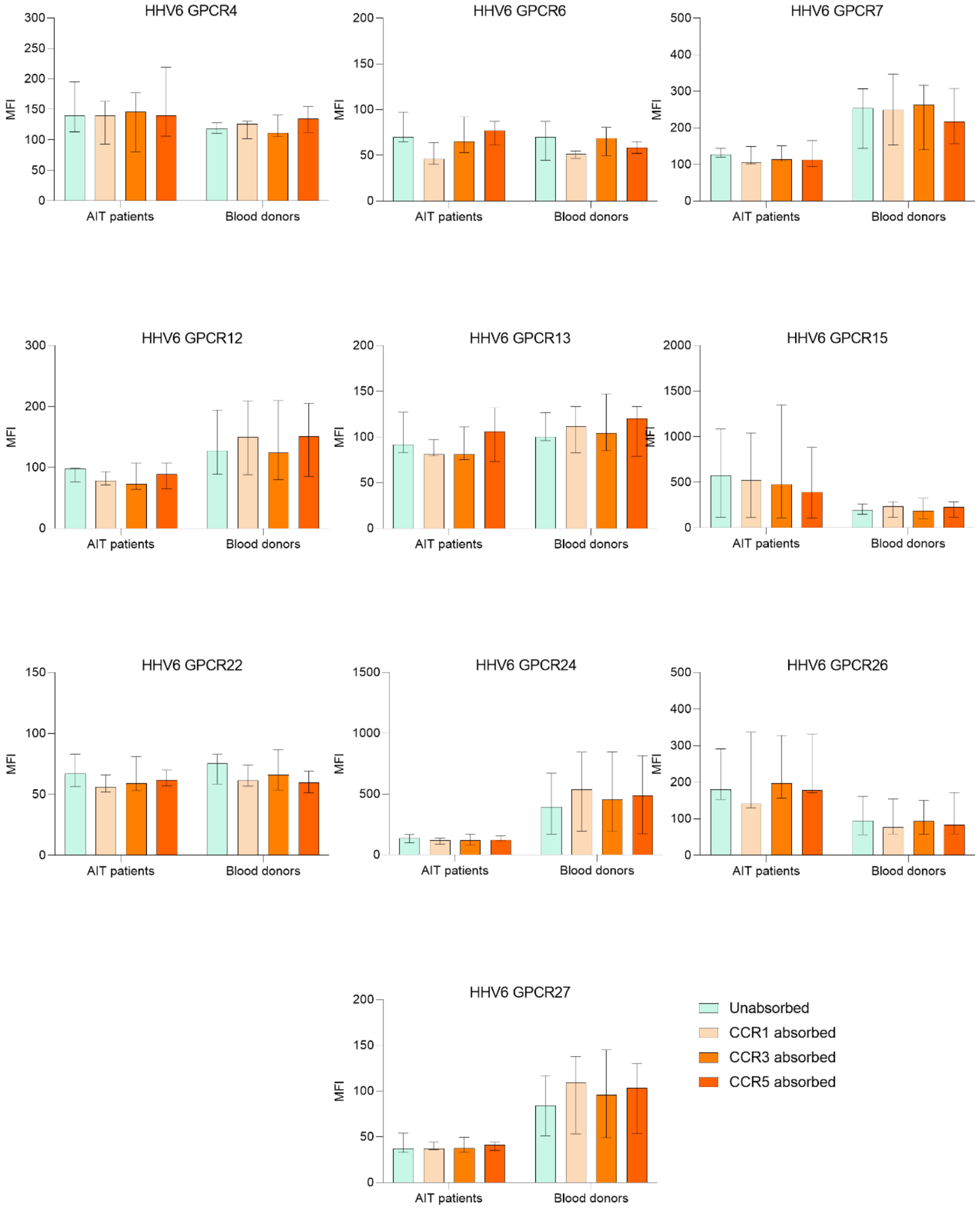

Supplementary figure 8. SMIA results of immunogenic peptides' IgG signals with and without human recombinant CCR1, CCR3, as well as CCR5 preabsorption

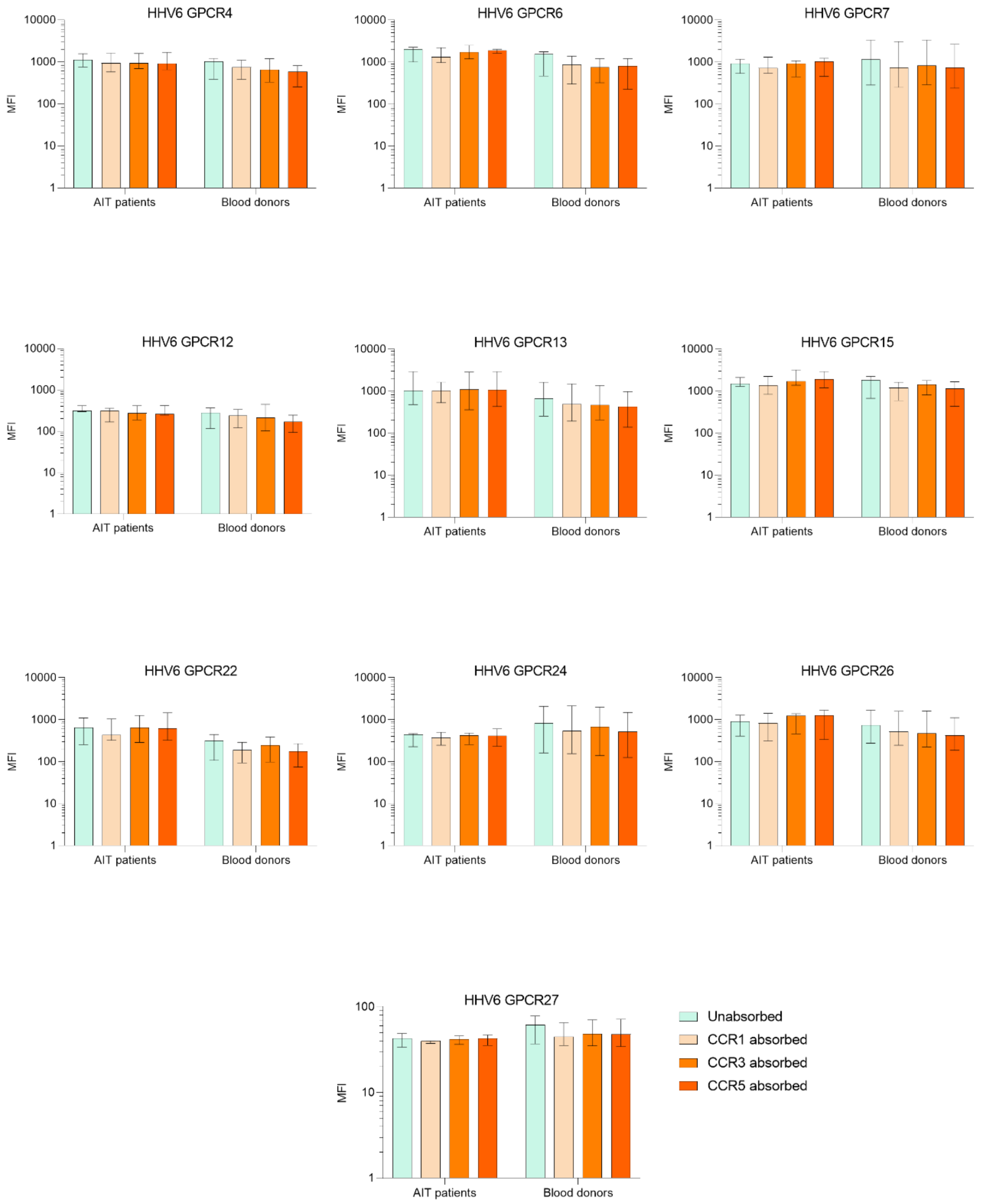

Supplementary figure 9. SMIA results of immunogenic peptides' IgM signals with and without human recombinant CCR1, CCR3, as well as CCR5 preabsorption.

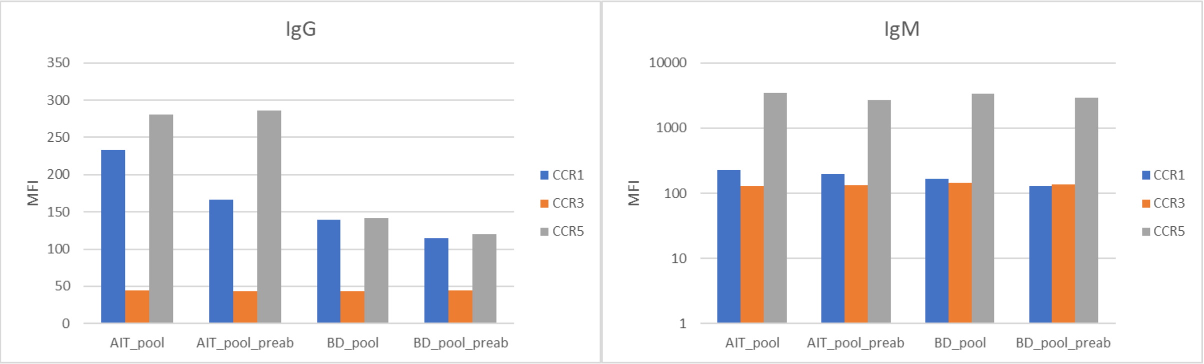

Supplementary figure 10. SMIA results of human recombinant CCR1, CCR3 and CCR5 IgG and IgM signals with and without reactive HHV-6 synthetic peptides preabsorption.
